# Underlying Differences in Health Spending Within the World Health Organisation Europe Region—Comparing EU15, EU Post-2004, CIS, EU Candidate, and CARINFONET Countries

**DOI:** 10.3390/ijerph16173043

**Published:** 2019-08-22

**Authors:** Mihajlo Jakovljevic, Paula Odete Fernandes, João Paulo Teixeira, Nemanja Rancic, Yuriy Timofeyev, Vladimir Reshetnikov

**Affiliations:** 1 Department of Global Health Economics and Policy, Faculty of Medical Sciences, University of Kragujevac, 34000 Kragujevac, Serbia; 2 Division of Health Economics, Lund University, SE 220 07 Lund, Sweden; 3 UNIAG, Applied Management Research Unit, Instituto Politécnico de Bragança, 5300-253 Bragança, Portugal; 4 CEDRI, Research Center in Digitalization and Intelligent Robotic, 5300-253 Bragança, Portugal; 5 Faculty of Medicine, University of Defence, 11000 Belgrade, Serbia; 6 Faculty of Business and Management, National Research University Higher Schools of Economics, 101000 Moscow, Russia; 7 Department for Health Care and Public Health, Sechenov First Moscow State Medical University, Trubetskaya, 8, 119048 Moscow, Russia

**Keywords:** health economics, WHO, Europe, public health expenditure, private health expenditure

## Abstract

This study examined the differences in health spending within the World Health Organization (WHO) Europe region by comparing the EU15, the EU post-2004, CIS, EU Candidate and CARINFONET countries. The WHO European Region (53 countries) has been divided into the following sub-groups: EU15, EU post-2004, CIS, EU Candidate countries and CARINFONET countries. The study period, based on the availability of WHO Global Health expenditure data, was 1995 to 2014. EU15 countries have exhibited the strongest growth in total health spending both in nominal and purchasing power parity terms. The dynamics of CIS members’ private sector expenditure growth as a percentage of GDP change has exceeded that of other groups. Private sector expenditure on health as a percentage of total government expenditure, has steadily the highest percentage point share among CARINFONET countries. Furthermore, private households’ out-of-pocket payments on health as a percentage of total health expenditure, has been dominated by Central Asian republics for most of the period, although, for the period 2010 to 2014, the latter have tended to converge with those of CIS countries. Western EU15 nations have shown a serious growth of health expenditure far exceeding their pace of real economic growth in the long run. There is concerning growth of private health spending among the CIS and CARINFONET nations. It reflects growing citizen vulnerability in terms of questionable affordability of healthcare. Health care investment capability has grown most substantially in the Russian Federation, Turkey and Poland being the classical examples of emerging markets.

## 1. Introduction

Healthcare spending became a matter of academic attention in the early works of the American economists Milton Friedman [1] and Selma Mushkin [2] ranging from the early 1920s until the early 1950s. These publications are counted among the historical beginnings of the interdisciplinary knowledge, which became known as the science of health economics [3]. Why did it all begin in the U.S., given the fact that early institutionalized national health systems had roots in Europe from the XIX (19th) century? In the U.S., since the 1840s up to the 1960s, according to available historical archives, there was a steady and parallel growth of the average industry worker’s net salary and the price of an ordinary physician examination [4]. After the 1950s, due to an array of socioeconomic changes and technology driven causes, these two trends became divergent leading to obvious disparity [5]. European nations, experiencing consecutive industrial revolutions were expanding their health care coverage within their nations very gradually. During the early years of Bismarck’s leadership, healthcare was provided only for industrial workers and their families from 1883 [6]. Beveridge evolved in the British mainland and its overseas colonies with some distinctive advantages being introduced around 1911 [7,8]. Surprisingly, the very first world’s healthcare system delivering universal health coverage for all its citizens including the poorest ones, at the level of medical technology of its time, was Soviet Semashko back in the early 1930s [9,10].

Today in Europe, North America and many other industrialized contemporary societies of Asia, real gross domestic product (GDP) growth is almost twice as slow as the long-term growth of total health expenditure [5]. The most commonly cited causes for health expenditure growth are advances in medical technology and pharmaceutical innovation [11], population aging [12], blossoming of non-communicable “prosperity” diseases [13] and growing citizen expectations [14]. A deeper understanding of long-term health expenditure patterns across geographies and jurisdictions might be essential for shaping responses to this challenge in the future [15].

This study aimed to reveal and explain the causes of underlying differences in health spending within the World Health Organization (WHO) Europe region by comparing the EU15, the EU post-2004, Commonwealth of Independent States (CIS) [16], EU Candidate and Central Asian Republics Information Network (CARINFONET) countries. This article contributes to the literature in several ways. Firstly, the studies on comparative research of healthcare systems have been rather limited to the Post-Soviet cultural space [15,17] outside the Russian language literature [18,19,20]. Secondly, it has added to the research on institutional change, which has been ongoing within the WHO Europe region since 1990 [21]. This concept of “transition” [22] from socialist centrally-planned economies, towards market-driven capitalist societies took place after the end of the Cold War era throughout the 1990s [23] and 2000s [24] alongside accelerated globalisation. This process had a profound impact on healthcare establishments [25], which is further explored alongside several distinctive development pathways.

Apparently, 45 years of the Cold War era [26] and consecutive accelerated globalisation [27] since 1991, has left a deep imprint on many spheres of life including healthcare systems [28]. Today, the major transnational associations on the continent are the European Union and the Commonwealth of Independent States as a legal successor authority of the Union of Soviet Socialist Republics (USSR) [29]. There are as well, other countries with various degrees of legislative proximity to one or another. The free-market or centrally-planned economic system establishment [30], prior to 1989 has had a profound impact on healthcare provision and financing patterns [25].

## 2. Materials and Methods

The geopolitical landscape within the WHO Europe Region comprising 53 countries and a total of 800+ million people was observed here by delineating specific groups of nations. Therefore, the WHO European Region was split into a total of five groups of nations (Table 1): EU15 (members of the European Union prior to 2004, having the established free-market economies since the Cold War era), the EU post-2004 (mostly former socialist, centrally-planned economies with the exceptions of Malta and Cyprus), the CIS (Commonwealth of Independent States with membership similar to 1991, due to the fact that all these countries shared the same Semashko healthcare systems), EU Candidate countries (consisting of former Yugoslavia republics/Western Balkans and Turkey) and CARINFONET countries (Central Asian nations, most of them members of the CIS, but represented a distinct subgroup of nations due to their shared socioeconomic characteristics.

The selected indicators of health spending were: total health expenditure as a percentage of Gross Domestic Product (GDP); public sector expenditure on health as a percentage of GDP; private sector expenditure on health as a percentage of GDP; total health expenditure, purchasing power parity, USD per capita; public expenditure on health, purchasing power parity, USD per capita; public sector expenditure on health as a percentage of total government expenditure; private households’ out-of-pocket payments on health as a percentage of total health expenditure. While the WHO and The Organisation for Economic Co-operation and Development (OECD) estimates were used for the first indicator, the WHO estimates were only utilized for the others.

The data source selected, among OECD Health, EuroStat, World Bank Health Data and a few other similar sources, was the WHO Global Health Expenditure Database due to its broadest possible coverage of political entities and highly reliable methodology of data collection giving insight into the exact fiscal flows within various national health systems [31]. The time horizon observed was 1995–2014 for most of the indicators, due to data gaps in chronology for the earlier years. For total health expenditure, expressed as a percentage of GDP, and public health spending, the time horizon was 1990–2014. Coverage of all observed nations, with these aggregate data, was complete and no significant data gaps were present.

Whenever possible, the OECD definition of total health expenditure was applied in this study (for details see [32], p. 89). It consisted of: household health expenses, including goods and services purchased at the consumer’s own initiative and the cost sharing part of publicly financed or supplied care; government-supplied health services, including those in schools, prisons and armed forces and special public health programs such as vaccination; investment in clinics, laboratories, etc.; administration costs; research and development, excluding outlays by pharmaceutical firms; industrial medicine; outlays of voluntary and benevolent institutions. In the case of most central and eastern European countries, the following had to be included: direct state budget allocated to the health sector, state subsidies to the mandatory health insurance system; mandatory health insurance contributions by employers and employees; direct health expenditure of employers for running industrial medical facilities; direct health expenditures of ministries and governmental agencies; charity health expenditures; foreign assistance; outstanding debt at the end of the year; private health insurance and direct private health charges [33]. It is important to ensure that funding from the general budget revenues and health insurance contributions do not overlap [34].

## 3. Results

The first part of the analysis reported in this section was related to the GDP of the five groups of nations. This data presented was estimated by the WHO.

**Figure 1:**
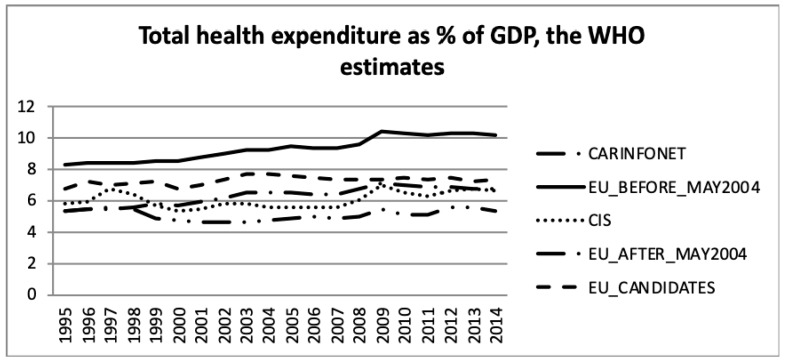
Total health expenditure as a percentage of Gross Domestic Product, the World Health Organization estimates.

**Figure 2:**
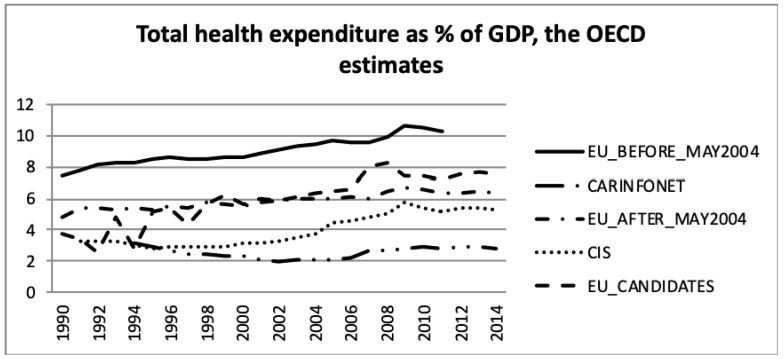
Total health expenditure as a percentage of Gross Domestic Product (GDP), the Organisation for Economic Co-operation and Development (OECD) estimates.

Figure 1 and Figure 2 present the total health expenditure as a percentage of GDP. Total health expenditures are the sum of general government expenditure on health and private expenditure on health.

The data of Figure 2, for OECD member states, was taken from the OECD Health Database [35]. For non-OECD countries, the data was as reported by the country in the European Health for All database (HFA-DB) [36] and may not have necessarily corresponded to the common WHO or OECD definition [37].

Comparing the health expenditure as a percentage of GDP, presented in Figure 1 and Figure 2, the data of EU_Candidates differed significantly between both sources. There was a gap in the data as shown in Figure 2 for the year 2011 and later for the EU_Before_May2004 group of nations. Nevertheless, both estimates showed that health expenditure was a greater share of GDP for the EU_Before_May2004 group of nations, and lower for the CARINFONET group. The EU_Before_May2004 group of nations had an increase in health expenditure as a percentage of GDP according to both data sources. Meanwhile, the EU_After_May2004, CIS and EU_Candidates also showed an increase in health expenditure as a proportion of GDP, but only in Figure 2, not confirmed by the WHO estimates (Figure 1).

**Figure 3:**
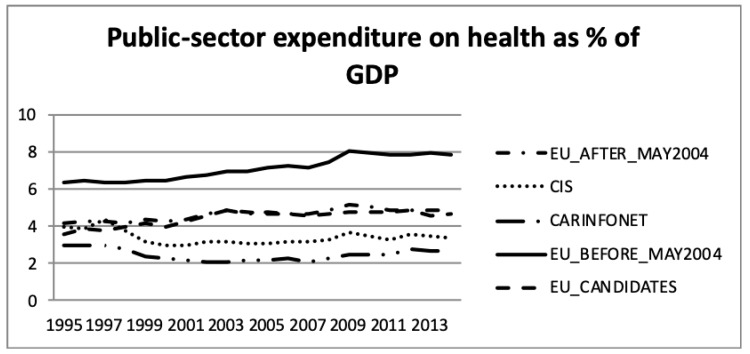
Public and private sector expenditure on health as a percentage of Gross Domestic Product (GDP), the World Health Organization estimates.

**Figure 4:**
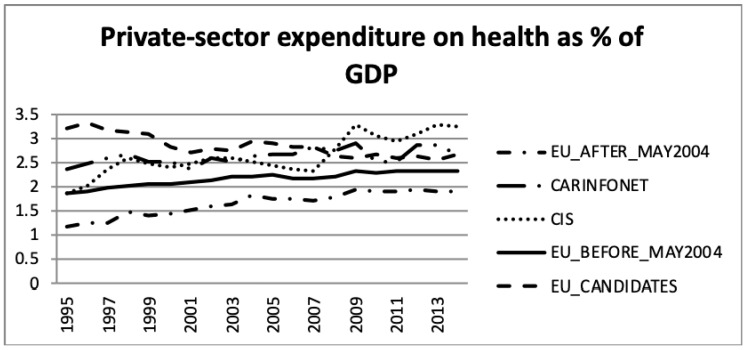
Public and private sector expenditure on health as a percentage of Gross Domestic Product (GDP), the World Health Organization estimates.

Concerning the comparison between public- and private sector expenditure on health as a percentage of GDP, Figure 3 and Figure 4 present the WHO estimates.

For the EU_Before_May2004 group of nations both public- and private sector expenditure on health, as a percentage of GDP increased by two and 0.5 percentage points, respectively. This was in accordance with the increase of the total health expenditure presented in Figure 1 and Figure 2. This group had the highest public sector expenditure and the second lowest private sector expenditure among the five groups of nations. However, this group of nations had an increase in investment until 2009, which stabilised after 2009.

The EU_After_May2004 group showed a similar tendency in public- and private sector expenditure on health, as a percentage of GDP, as the other EU nations. Health expenditure, however, accounted for a lower proportion of GDP for this group of countries.

These data show that the EU member nations—both those who were members before and after May 2004—experienced increasing health expenditure GDP shares until 2009, but thereafter health expenditure accounted for a stable share of the economy. A comparison of Figure 1 and Figure 2 shows that the public sector health expenditure of each group was three to four times larger than the respective private expenditure share.

Concerning the CIS and CARINFONET groups of nations, they presented a similar pattern in the total health expenditure, as a percentage of GDP, both in public and private sectors. Nevertheless, the CARINFONET had a generally lower investment in healthcare, and in public spending, of about one point in percentage point share terms. Concerning the private investment, they presented a similar level of investment (by the percentage of GDP). However, the CIS group significantly increased their private investment in 1995–1998 and 2008–2009. The CIS group of nations (which included the CARINFONET group) had a stabilised level of total investment (about 6% of GDP). The public sector expenditure remained stabilised between 3% and 4% of GDP, meanwhile the private sector expenditure increased from 2% to more than 3% of GDP. The public and private expenditure appeared to be balanced under the CIS group of nations.

Concerning the EU_Candidate countries, the public sector expenditure on health remained stable, but the private sector suffered a decrease of almost one percentage point from 3.2% to 2.5% of GDP. This trend of lower expenditure in the private sector, among the EU Candidate economies, was opposite as a tendency with regard to the other groups of nations. The EU_Candidates private expenditure seemed to follow an approximation to the level of private expenditure of the EU nations.

**Figure 5:**
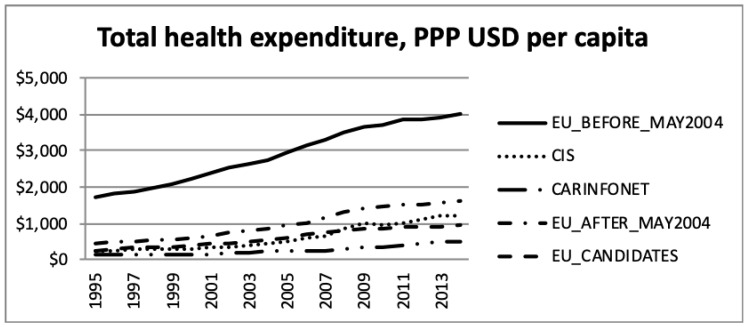
Total health expenditure, purchasing power parity (USD) per capita, the World Health Organisation estimates. Note:

**Figure 6:**
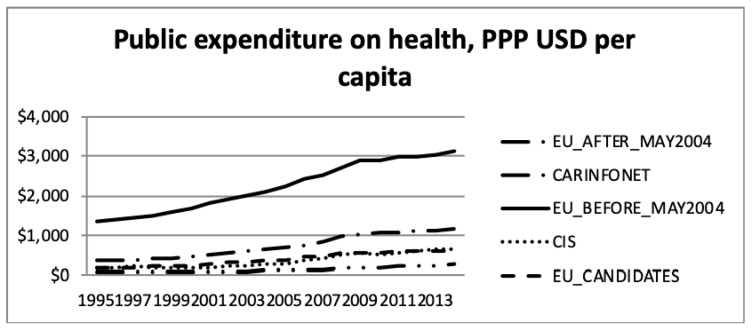
Public health expenditure, purchasing power parity (USD) per capita, the World Health Organisation estimates.

The total health expenditure, purchasing power parity (PPP), in USD per capita, and the public sector expenditure on health, purchasing power parity, in USD per capita, according to the WHO estimates, is presented in Figure 5 and Figure 6.

The data presented in Figure 5 and Figure 6 are net values, not considering the GDP and inflation, during the period. Therefore, this increase should be read under this consideration. Thus, the increase in total and public sector expenditure on health during the period 1995 to 2013, should be regarded considering the inflation. The different level of expenditure among groups of nations showed a significantly higher expenditure for EU Members prior to May 2004. However, this difference should be regarded in the face of different levels of costs, among the groups of nations, for the same type of operation/product. In this group the price of medical care was generally higher than in the other groups of nations.

In absolute nominal monetary terms, Western European EU15 countries have exhibited the strongest upward growth in total health spending. Observing the purchasing power parity landscape [38], the EU15 nations were mostly outperforming their counterparts in Eastern Europe, the Balkans and Central Asia, not only in the public sector, but also in total.

**Figure 7:**
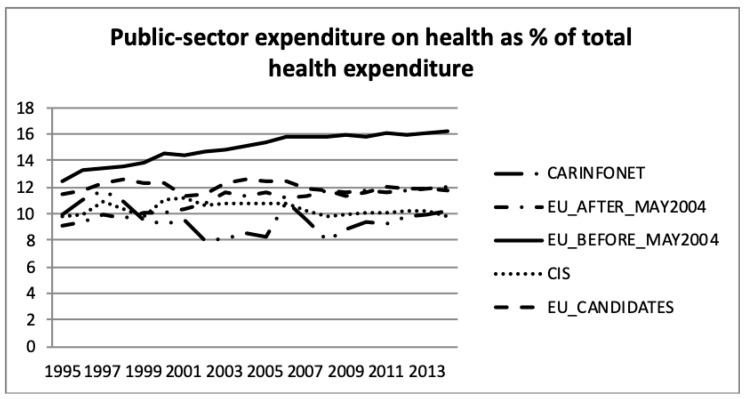
Public sector expenditure on health as a percentage of total health expenditure, the World Health Organisation estimates.

**Figure 8:**
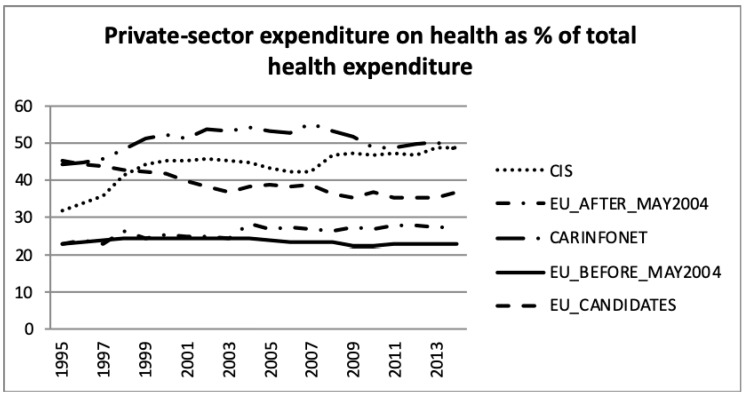
Private sector expenditure on health as a percentage of total health expenditure, the World Health Organisation estimates.

Figure 7 and Figure 8 present the public- and private sector expenditure on health, as a percentage of total health expenditure, according to the WHO estimates. This information was in accordance with public sector expenditure on health, as a percentage of GDP, presented in Figure 2, but showed the government expenditure in public sector health. It can be seen that the EU_Before_May2004 group of nations had a higher investment in health than the other groups. The EU_Before and After_May2004 had an increase until 2009, but after that it remained stable until 2013, about 16% for EU_Before_May2004 and 12% for EU_After_May2004. The EU_Candidates aligned the public sector expenditure on health with EU_After_May2004 since 2008. The CIS group of nations had a stabilised effort on expenditure in the public health sector of around 10% of total government expenditure. The CARINFONET group of nations had several fluctuations of public sector expenditure between 1995 and 2013.

**Figure 9:**
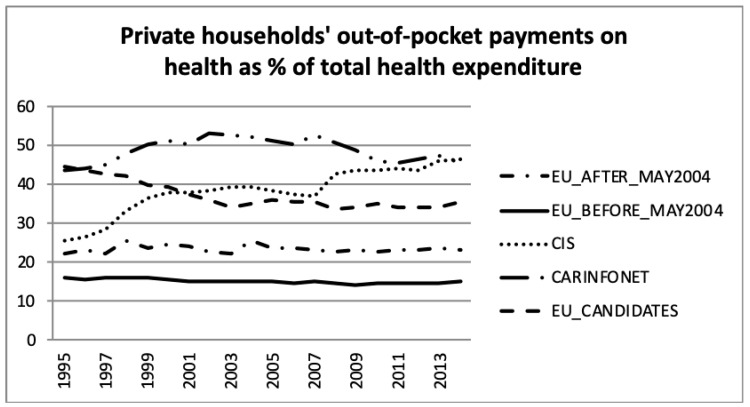
Private households’ out-of-pocket payments on health as a percentage of total health expenditure, the World Health Organisation estimates.

Figure 9 presents the private households’ out-of-pocket payments on health as a percentage of total health expenditure for the five groups of nations. This feature was dominated by the Central Asian republics (CARINFONET) for most of the period, shared with CIS values in latter years. The EU_Before_May2004 and EU After_May2004 presented a lower and stable level of private households’ out-of-pocket spending on health, which was around 15% and 23% of total health expenditure, respectively. These results were in accordance with previous analysis of Figure 4, Figure 5 and Figure 6. The EU_Candidates’ group of nations made an effort to reduce its expenditure in this category and stabilised after 2003, to around 33% to 36% of total health expenditure.

## 4. Study Limitations

One of the possible methodological weaknesses of this study is the fact that reported annual values of health spending by the major multilateral agencies such as the World Bank (WB) and OECD Health may differ to a minor extent (up to +/− few percentage point shares). Geoeconomic division of WHO European region into the five observed regions might be questioned to some extent, alongside other major division lines separating diverse health financing and provision traditions across Europe. One such possible division that might be objected to as an alternative core criterion is the separation of the European health systems to Bismarck, Beveridge and Semashko route models, following their historical order of appearance. Estimates for total expenditure on health produced by the WHO were reliable to the greatest extent possible, based on the National Health Accounts [39] classification [40]. The sources included both nationally reported data and estimates from international organisations namely: WHO, the United Nations (UN), the International Monetary Fund (IMF), WB and OECD. Therefore, they may differ somewhat from official national statistics reported by countries [41]. The WHO estimates for this indicator are generally more accurate [42]. Therefore, the authors worked with the best publicly accessible data for international comparisons of inherently different economic systems. However, the core findings of the study should not be questioned based on these minor differences in accounting practices across jurisdictions.

## 5. Discussion

The historical legacy of the Cold War era and accelerated globalisation in the post-1989 period, has shaped economic systems throughout the European continent. During this process, several distinctive geopolitical [43] and economic zones have emerged [44,45]. This research has explored the best publicly accessible international data sources on the long-term evolution of healthcare-related spending. Comparison of these five groups of nations, sharing significant internal similarities, reveals surprising findings which so far, remain unpublished in literature [46].

Since a lengthy time period was explored, there were roughly two different stages of historical evolution particularly prominent on the eastern side of the former Iron Curtain frontier. For most of the 1990s the entire Eastern Europe, being mutually interdependent with the former USSR, was dragged into the new Russian recession, bottoming out in 1998. This heavily reflected on demographic trends of the time, healthcare investment and its outcomes in most of the former socialist countries. This early transitional period was marked with difficulties, even with quite successful health reforms, such as the Polish [47], Hungarian [48] or Czech ones [49]. In the civil wars of the former Yugoslavia, sanctions and waves of refugee migration devastated economies and led to a decade-long delay in significant health reforms and spending growth [50]. This was most prominent in the largest country of the region, Serbia [51], acting as the historical core for Yugoslavia after World War I and World War II [12]. Turkey had a rather slower pace of essential health system progress back in the 1990s [52].

The second, far more prosperous period, roughly began in the early 2000s, for most of the Eastern European [53,54] and Balkan nations [55]. Since the beginning of the XXI (19th) century, just like the Russian Federation’s [9], Turkey’s overall development and ability to increase health expenditure was growing much faster in comparison to the high-income OECD economies [56]. The contemporary Turkish health system delivered surprisingly well-balanced, and, to a large degree, cost-effective spending mechanisms, leading to limited inequalities and medical care affordability [57].

The high-income Western European EU15 nations had rather steady and satisfactory, overall economic growth spanning from 1989 to 2007 [58]. However, between 1990 and 2014, these rich countries exhibited an exceptionally alarming rate of health spending growth, even when their real GDP growth was taken into account [59]. This means that public cost containment policies, conducted over the past thirty years, have been largely ineffective in dealing with this challenge [60]. Accelerated and advanced stage population aging [61] remains particularly prominent in Western, Northern and Central Europe [62]. One should emphasize here that this core driver of health-spending remains slightly less influential in Eastern European societies compared to EU15. This is due to the fact that population-aging evolution, in Eastern Europe, is at an earlier stage, as was recently documented in UN datasets, comprising data from the previous century [63]. Furthermore, the Russian Federation’s early 2000s fertility-supportive policies gained momentum with a recent RANEPA report [64] claiming total fertility growth rate [65], on average, from 1.3 to 1.7 children per woman, over a decade long perspective [64]. Another fact is that life expectancies remain significantly lower across the CIS nations [66] with men living shorter lives than women [67]. However, exploring the core investment–output ration between health spending and longevity, throughout the vast Eastern EU-post-2004, the Balkans and CIS region provide another surprising perspective. Life expectancy gains in South-East Europe [68] were quite similar to EU-post-2004 members at a far lower healthcare investment increase [69]. Furthermore, the CIS relationship curve, between current total health expenditure and longevity growth margin, although positioned in the lower quadrant, in comparison to the other two regions, has the steepest upward pathway, promising improvement in the future [70].

Therefore, it seems that the EU nations (before and after May 2004), had an increase of expenditures as a percentage of GDP until 2009, but stopped the further growth after this year [71]. This is clearly attributable to the fiscal austerity policies triggered by the Global Recession, following the bankruptcy of the Lehman Brothers in 2007 [72]. Such strategies were centrally designated by the European Commission among EU members [73] and EU Candidate countries to a lesser extent [74]. The lasting macroeconomic crisis effects, between 2007 and 2016, contributed significantly to the worsening of health financing sustainability. A recent Brookings Institute report, based on World Bank data, claims the beginnings of sustainable global economic growth in 2017 and 2018 [75]. Their estimates are that one half of this real GDP growth, worldwide, remains to be driven by seven major Emerging Markets or EM7 (BRICS + Mexico, Indonesia and Turkey), while only a quarter by G7 nations on the time horizon 2017–2020 [76]. These promising signs give hope for a European-wide ability to invest more in health, which should be recovered in the near future. This refers to not only nominal and purchasing power parity spending, but also percentage point share of real disposable GDP.

## 6. Conclusions

Regardless of some grounds for optimism, there is a prevailing consensus among health policy authorities in the EU and Eurasian Union, CIS and WHO Europe alike, that health financing efficiency will have to be substantially improved. The core underlying assumption of all European health system establishments, since their late XIX century creation, was a demographic growth model. This postulate has now changed in Europe and worldwide, with the advent and acceleration of global population aging. A shrinking workforce and contracted tax base in the labor market are constraining the supply-side of modern-day societies’ ability to invest in medical care. The demand-side is burdened by the spreading of non-communicable diseases, demand for long-term, home-based care, family caregiving and prohibitively expensive last-year-of-life, amounting to the entire lifetime medical consumption of a citizen. Given these core drivers in the equation, technological innovation in medicine, and consecutive public demand for cutting-edge technologies remain far less containable areas by cost-effective resource allocation policies. These challenges, alongside fiscal constraints, are shared across the East and West of the European continent in the long run. Hopefully, these vast regions will be able to learn from each other’s distinctive historical experiences and create more adaptive system responses in the future.

## Figures and Tables

**Table 1:** List of country groups observed.

EU15	EU Post-2004	CIS *	CARINFONET **	EU Candidate Countries
Austria AUT	Bulgaria BGR	Armenia ARM	Kazakhstan KAZ	Albania ALB
Belgium BEL	Croatia HRV	Azerbaijan AZE	Kyrgyzstan KGZ	Montenegro MNE
Denmark DNK	Cyprus CYP	Belarus BLR	Tajikistan TJK	Serbia SRB
Finland FIN	Czech Republic CZE	Kazakhstan KAZ	Turkmenistan TKM	The former Yugoslav Republic of Macedonia MKD
France FRA	Estonia EST	Kyrgyzstan KGZ	Uzbekistan UZB	Turkey TUR
Germany DEU	Hungary HUN	Republic of Moldova MDA		Bosnia and Herzegovina BIH
Greece GRC	Latvia LVA	Russian Federation RUS		
Ireland IRL	Lithuania LTU	Tajikistan TJK		
Italy ITA	Malta MLT	Turkmenistan TKM		
Luxembourg LUX	Poland POL	Ukraine UKR		
Netherlands NLD	Romania ROU	Uzbekistan UZB		
Portugal PRT	Slovakia SVK			
Spain ESP	Slovenia SVN			
Sweden SWE				
United Kingdom GBR				

Note: * The Commonwealth of Independent States (CIS) formed when the former Soviet Union dissolved in 1991. ** CARINFONET (Central Asian Republics Information Network) Steering Group.
